# A Nationwide Survey on Danon Disease in Japan

**DOI:** 10.3390/ijms19113507

**Published:** 2018-11-08

**Authors:** Kazuma Sugie, Hirofumi Komaki, Nobuyuki Eura, Tomo Shiota, Kenji Onoue, Hiroyasu Tsukaguchi, Narihiro Minami, Megumu Ogawa, Takao Kiriyama, Hiroshi Kataoka, Yoshihiko Saito, Ikuya Nonaka, Ichizo Nishino

**Affiliations:** 1 Department of Neurology, Nara Medical University School of Medicine, Nara 634-8521, Japan; neura@naramed-u.ac.jp (N.E.); shiotatomo@naramed-u.ac.jp (T.S.); kirinara@yahoo.co.jp (T.K.); hk55@naramed-u.ac.jp (H.K.); 2 Department of Neuromuscular Research, National Institute of Neurology, National Center of Neurology and Psychiatry, Tokyo 187-8502, Japan; minami@ncnp.go.jp (N.M.); ogawa@ncnp.go.jp (M.O); nishino@ncnp.go.jp (I.N.); 3 Department of Child Neurology, National Center Hospital, National Center of Neurology and Psychiatry Hospital, Tokyo 187-8502, Japan; komakih@ncnp.go.jp (H.K.); nonakai@za.cyberhome.ne.jp (I.N.); 4 Department of Cardiology, Nara Medical University School of Medicine, Nara 634-8521, Japan; konoue@naramed-u.ac.jp (K.O.); yssaito@naramed-u.ac.jp (Y.S.); 5 Department of Nephrology, Kansai Medical University, Osaka 573-1010, Japan; tsukaguh@hirakata.kmu.ac.jp

**Keywords:** Danon disease, lysosome-associated membrane protein-2 (LAMP-2), hypertrophic cardiomyopathy, myopathy, autophagy, heart transplantation, cerebral infarction, autophagic vacuoles with sarcolemmal features (AVSF), Wolf–Parkinson–White (WPW) syndrome

## Abstract

Danon disease, an X-linked dominant cardioskeletal myopathy, is caused by primary deficiency of lysosome-associated membrane protein-2 (LAMP-2). To clarify the clinicopathological features and management, we performed the first nationwide, questionnaire-based survey on Danon disease in Japan. A total of 39 patients (17 males, 22 females) from 20 families were identified in the analysis. All patients had cardiomyopathy. Of the 21 patients who died, 20 (95%) died of cardiac failure or sudden cardiac arrest. Most patients had hypertrophic cardiomyopathy. Wolf–Parkinson–White syndrome was present at a comparatively high incidence (54% in males, 22% in females). Only one female patient received a heart transplant, which is the most effective therapy. Histopathologically, all male patients showed autophagic vacuoles with sarcolemmal features in muscle. Half of the probands showed de novo mutations. Male patients showed completely absent LAMP-2 expression in muscle. In contrast, female patients showed decreased LAMP-2 expression, which is suggested to reflect LAMP-2 haploinsufficiency due to a heterozygous null mutation. In conclusion, Danon disease is an extremely rare muscular disorder in Japan. Cardiomyopathy is the most significant prognostic factor and the main cause of death. Our findings suggest that the present survey can extend our understanding of the clinical features of this rare disease.

## 1. Introduction

Danon disease (MIM #300257) is an X-linked dominant cardioskeletal myopathy caused by a primary deficiency of lysosome-associated membrane protein-2 (LAMP-2) [1,2]. LAMP-2 is a highly glycosylated protein present on the inside of the lysosomal membrane. Although the precise functional role of LAMP-2 remains controversial, the LAMP-2 protein is a key molecule in the final process of autophagy that mediates the fusion between autophagosomes and lysosomes. We previously reported the first review article on the clinical features of 20 patients from 13 families with genetically confirmed Danon disease in the world [2]. We revealed that Danon disease was clinically characterized by the triad of cardiomyopathy, myopathy, and mental retardation in males, but only cardiomyopathy in females [1,2,3,4,5]. Cardiac involvement, especially hypertrophic cardiomyopathy, was the most principal and coherent manifestation in both male and female patients with Danon disease and is diagnostically very significant. Interestingly, pathological studies of muscle have revealed autophagic vacuoles with sarcolemmal features (AVSF) in male patients [6,7]. AVSF with acetylcholinesterase activity refers to the unique, disease-specific autophagic vacuoles that delineate Danon disease and related myopathies such as X-linked myopathy with excessive autophagy.

However, Danon disease is an extremely rare disease, and very few cases have been reported. We considered that there were a certain number of patients in whom Danon disease was underdiagnosed owing to diagnostic difficulties or unawareness of the disease. Therefore, the clinicopathological features and management of Danon disease have not been well established. In this study, we performed a nationwide, questionnaire-based survey on Danon disease in Japan to elucidate its clinical characteristics. In addition, this survey was designed to clarify the previously unreported longitudinal histories of patients with definite Danon disease.

## 2. Results

### 2.1. Patients

The first set of questionnaires was returned from 1409 departments or hospitals (response rate 53.8%). The second set of questionnaires was sent to a total of 21 departments or hospitals that had clinical information on patients with definite and suspected Danon disease. The detailed clinical records that were returned to us were carefully reviewed. In addition, a systematic literature review was conducted and yielded 15 articles or society reports [2,8,9,10,11,12,13,14,15,16,17,18,19,20,21,22].

**Figure 1:**
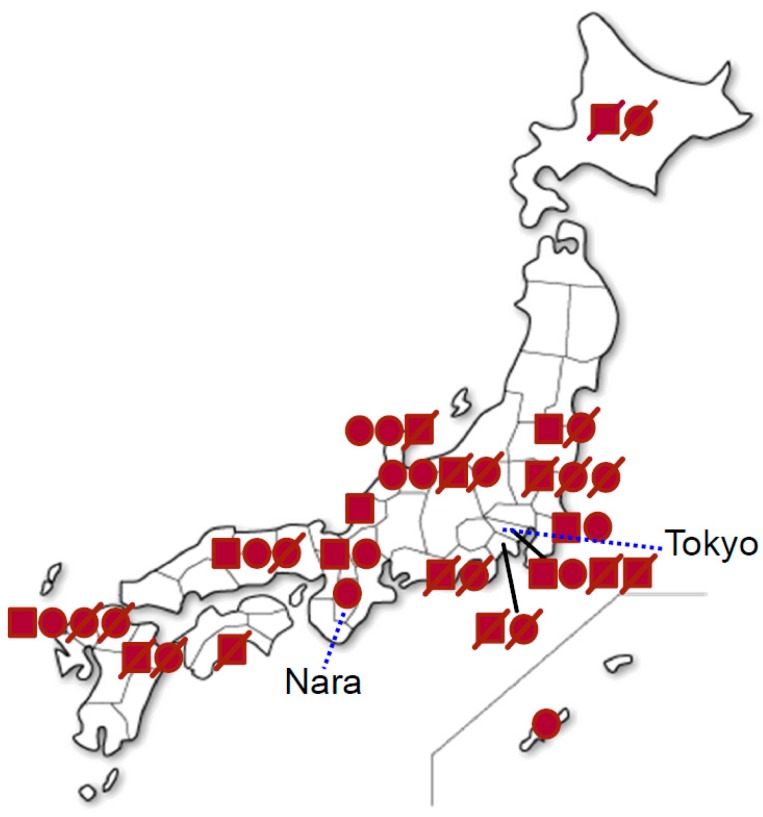
Distribution of the patients with Danon disease in Japan. Closed squares indicate male patients, whereas closed circles indicate female patients. Diagonal lines indicate dead patients.

As a result of the first nationwide survey, we identified 39 patients with Danon disease (17 males and 22 females) from 20 families (Table 1). Eighteen of these patients (7 males, 11 females) from 12 families were still alive at the time of the survey. The location of the patients was broadly distributed from the north to south in Japan (Figure 1).

### 2.2. Clinical Features

The clinical features of 17 male and 22 female patients are summarized in Table 2. Of the 20 families, 16 probands were male and four probands were female. Ten of the 20 mothers were symptomatic (50%). The three common features in males were cardiomyopathy, myopathy, and mental retardation, whereas the common feature in females was cardiomyopathy.

All patients with available information were born through normal pregnancy and delivery. Ages at onset varied from 1 to 19 years in the 17 male patients, and from 6 to 36 years in seven female patients. However, disease onset may be earlier, but go undetected because of the subacute nature and slow progression of the disease in male patients, while it is difficult to define the age at onset due to the insidious nature of the disease in adult female patients. Among the 21 patients (9 males and 12 females) who had died, 20 (95%) died of cardiac failure or sudden cardiac arrest, which is the main cause of death among patients. The other male patient died of juvenile transverse colon cancer. The mean age at death was 19 ± 5 years with a range of 13 to 26 years in seven male patients and 37 ± 11 years with a range of 20 to 52 years in seven female patients. No male patient survived beyond 26 years of age except for a 50-year-old man in Family 1. He had mild cardiomyopathy, but reported no cardiac symptoms.

All 17 male patients had cardiomyopathy. Most had hypertrophic cardiomyopathy, but two (12%) had dilated cardiomyopathy. All but one of the 22 female patients had cardiomyopathy, cardiac involvement, or both. Eleven (50%) had hypertrophic cardiomyopathy, six (27%) had dilated cardiomyopathy, and four (18%) had sudden cardiac arrest. Only one female patient (5%) had no cardiac involvement, although her monozygotic twin had hypertrophic cardiomyopathy [13]. Heart transplantation, the most effective therapy, was performed in only one female patient [17] and is currently required by four patients. Several patients were managed with left ventricular assist devices (LVAD), permanent pacemakers, and/or implantable cardioverter defibrillators.

Myopathy was usually mild and was observed in all male patients. While 13 (76%) of the 17 male patients showed muscle weakness, the other four patients (24%) had no muscle weakness with mild myogenic changes on electromyography. In contrast, myopathy was observed in two (9%) of 22 female patients. The main symptoms were mild proximal weakness in proximal limb or neck muscles. All male and female patients remained ambulatory. Mild mental retardation was present in eight (46%) of the 17 male patients and in two (9%) of the 22 female patients. On physical examination, retinopathy was observed in one male patient and one female patient. A few patients showed hepatomegaly, splenomegaly, and foot deformities, such as pes cavus. Two female patients had cerebral infarction. One female patient had young-onset stroke due to diffuse narrowing of the cerebral arteries without atherosclerosis, while the other had minor right cerebral infarction occurring after the LVAD operation.

Laboratory tests showed that the serum creatine kinase (CK) level increased to 5- to 10-fold above the upper limits of normal. The CK value was 1086 ± 715 IU/L with a range of 77 to 2865 in all male patients and 148 ± 164 IU/L with a range of 40 to 643 in 12 female patients. Electrocardiographic (ECG) findings were abnormal in all the patients tested. The most common pathological finding was Wolff–Parkinson–White (WPW) syndrome. Nine (54%) of 17 males and four (22%) of 18 females showed WPW syndrome. Male patients with hypertrophic cardiomyopathy showed abnormally high voltage in precordial leads. In addition, giant negative T waves, atrioventricular block, atrial flutter, atrial fibrillation, bradycardia, abnormal Q waves, and complete left bundle branch block were also observed. The echocardiography results show that most patients had concentric hypertrophic cardiomyopathy with impaired left ventricular function. Nine male patients had an abnormally thick interventricular septum and posterior walls. Electromyography showed small-amplitude short-duration motor unit potentials in all 11 male patients tested. In addition, fibrillation and positive sharp waves at rest were present. Electroencephalography (EEG) showed mild abnormalities in two male patients; one had moderate bitemporal slowing and the other had a slow α wave pattern with emergence of diffuse θ waves during sleep. Nerve conduction studies showed mild sensory and motor polyneuropathy in one (8%) of 13 patients tested.

### 2.3. Histochemistry and Immunohistochemistry

**Figure 2:**
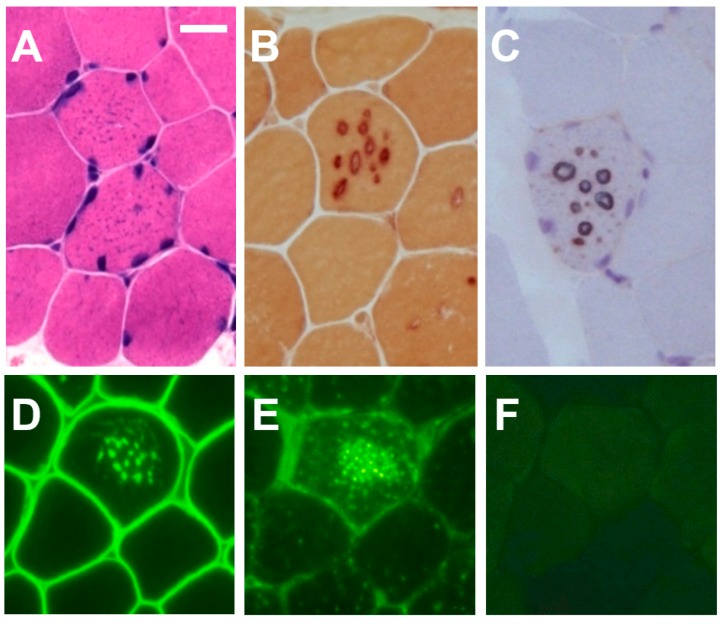
Muscle pathology of a male patient with Danon disease. Tiny autophagic vacuoles look like solid basophilic granules in muscle fibers with hematoxylin and eosin stain (**A**). The vacuolar membrane has non-specific esterase activity (**B**) and acetylcholinesterase activity (**C**). Immunohistochemical analyses for dystrophin (**D**) showed that the vacuolar membrane has features of sarcolemma. Immunostaining for LIMP-1 demonstrated the overexpression of the LIMP-1 protein (**E**), whereas immunostaining for LAMP-2 clearly demonstrated the complete absence of the LAMP-2 protein (**F**). Bar = 30 μm.

Pathologically, muscle biopsy specimens in male patients tested showed mild to moderate variations in fiber size (Figure 2). Small vacuoles were seen in many fibers, which may appear as basophilic granules rather than vacuoles in hematoxylin and eosin preparations. The granules contained acid phosphatase-positive material. In all male patients, acetylcholine and nonspecific esterase activities were associated with the vacuolar membranes. As the vacuolar membranes stained with antibody against dystrophin on immunohistochemical analysis, all male patients showed AVSF in muscles. LAMP-2 was completely absent in muscle from all male patients.

In contrast, female patients showed no variations in fiber size and no vacuoles. The acetylcholinesterase and non-specific esterase staining appeared normal. In addition, immunohistochemistry for LAMP-2 did not reveal a complete absence, but the reaction was weaker than that in normal controls. The other immunohistochemical studies revealed no abnormalities and no AVSF.

### 2.4. Electron Micsroscopy

**Figure 3:**
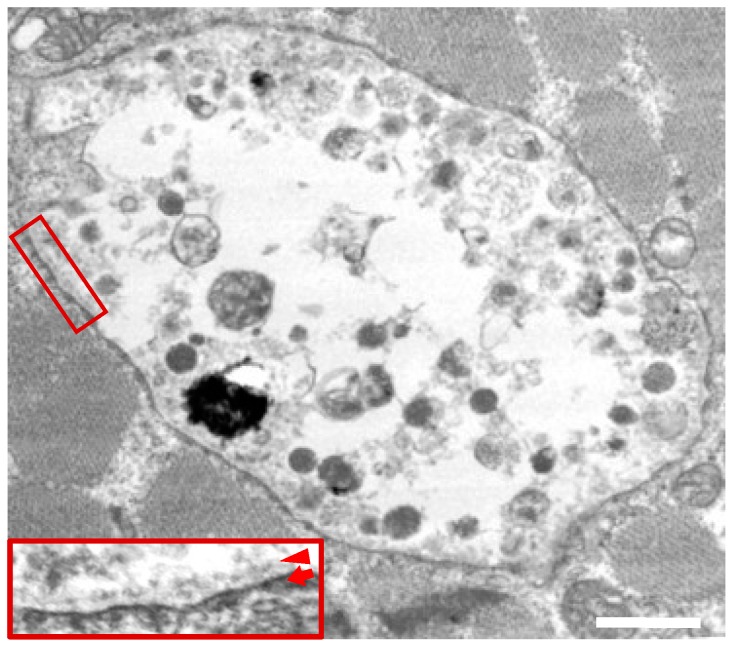
Electron microscopy in skeletal muscles from a male patient with Danon disease. The vacuoles had autophagic nature as indicated by the presence of electron-dense granular materials, myeloid bodies, and variable cytoplasmic debris. In addition, basal lamina (arrowhead) are likely to be observed along the inner surface of an autophagic vacuole (arrow). The inset reveals the enlarged view of the area within the square (magnification 4×). Reconstructed with permission from our previous report [6]. Bar = 1 nm.

The electron microscopy results showed that clusters of intracytoplasmic autophagic vacuoles containing electron-dense granular materials, myeloid bodies, and variable cytoplasmic debris were scattered in muscle (Figure 3). Some of these autophagic vacuoles were bounded by membranes on the luminal side, while other clusters were not limited by a membrane, as previously reported [6].

### 2.5. Western Blot Analysis

**Figure 4:**
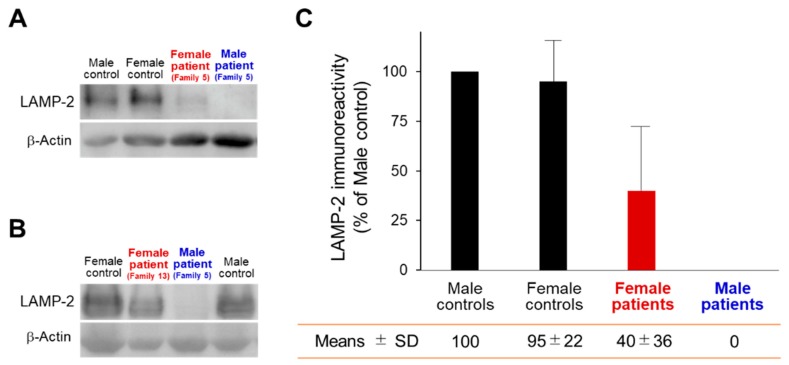
Western blot analysis in patients with Danon disease. (**A**,**B**) Skeletal muscle extracts from a male control and a female control, and the male patient and female patient, who were blotted and labeled with antibodies against LAMP-2 and β–actin. (**A**) The male patient and female patient from Family 5; (**B**) the male patient from Family 5 and the female patient from Family 13, reconstructed with permission from our previous reports [8,9]. (**C**) The relative protein expression levels in the male controls were expressed as 100%. LAMP-2 immunoreactivity was present equally in the both male and female control muscles. In contrast, LAMP-2 was undetectable in the male patients’ muscle, whereas two female patients showed decreased LAMP-2.

The relative protein expression levels in the male controls were 100% (Figure 4). On Western blot analysis, LAMP-2 immunoreactivity was present equally in the both male (100%) and female (95%) control muscles, as reported previously [8,9]. In contrast, LAMP-2 staining was completely absent (0%) in the male patients’ muscles, whereas two female patients in Family 5 and Family 13 showed decreased LAMP-2 staining (40%), most likely due to ‘LAMP-2 haploinsufficiency’, as reported previously [8,9].

### 2.6. Sequence Analysis of LAMP-2

**Figure 5:**
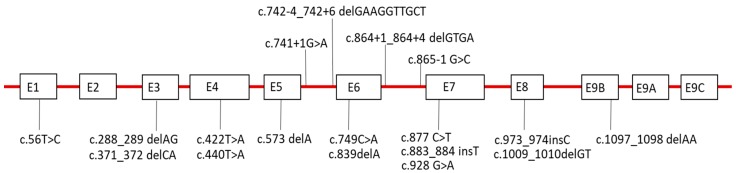
Schematic structure of the *LAMP-2* gene with the mutations identified in the present survey in Japan. Exons are in scale and introns are not to scale. E: Exon.

Mutations in *LAMP-2* were identified in all probands from the 19 families except for Family 17 (Figure 5, Table 1). As LAMP-2 was completely absent in muscle from the proband in Family 17, the family was finally given a diagnosis of Danon disease [20]. X chromosomes were heterozygous in all 19 families. Only Family 11 and Family 15 showed the same mutation (c.877C > T), but each of the other families had 17 different mutations. The 16 mutations had previously been reported as a novel mutation, while the other two mutations (c.877C > T, c.973_974insC) had already been reported. Although the distribution of mutations widely ranged from exon 1 to exon 9, six families (30%) had mutations associated with exon 7. All mutations were either nonsense or frame-shift mutations that are predicted to cause truncation of the protein, except for the exon 6 skipping mutation in Family 5. On screening 100 Japanese controls, the same mutations were not found. The mothers, family members, or both, had no mutations in *LAMP-2* in the probands from eight families.

## 3. Discussion

We conducted the first nationwide, questionnaire-based survey in Japan to clarify the prevalence, clinical features, muscle pathological findings, and mutation status of patients with Danon disease. We identified a total of 39 patients, 17 males and 22 females, from 20 families with definite Danon disease. All probands from 19 families had *LAMP-2* mutations. As the proband from the other family had LAMP-2 deficiency in muscle specimens, the family was finally given a diagnosis of Danon disease. In our previous global studies [2], we confirmed that the triad of cardiomyopathy, myopathy, and variable mental retardation encompasses the main clinical features in male patients, whereas only cardiomyopathy is found in female patients. In the present survey, we demonstrated that the same was true for the main clinical features in Japan.

Cardiac manifestations are prominent clinical features and the most significant prognostic factors, as most of the deceased patients died of cardiac failure [23,24,25]. Histologically, cardiomyocytes had autophagic vacuoles including myofibrillar disruption. Hypertrophic cardiomyopathy developed in most male patients and many female patients. Although some female patients showed dilated cardiomyopathy, we guessed that some patients had a dilated phase of hypertrophic cardiomyopathy. In fact, previous reports have described the evolution of hypertrophic cardiomyopathy into dilated cardiomyopathy with progressive cardiac failure. Therefore, dilated cardiomyopathy may be associated with the timing of cardiac investigations. The WPW ECG pattern observed in Danon disease has been attributed to myocardial hypertrophy rather than to the presence of an accessory pathway [26]. However, the incidence of WPW is relatively high (54% in males, 22% in females) in Danon disease as compared with that in other types of hypertrophic cardiomyopathy, such as idiopathic hypertrophic cardiomyopathy (1.5%) and familial hypertrophic cardiomyopathy (12%) [27,28]. This finding suggests the presence of a specific pathomechanism for preexcitation in Danon disease rather than a simple association with cardiac hypertrophy. In addition, more recently, it was reported that troponin I might have a prognostic value and merits exploration for clinical decisions [29]. Therefore, we expect that troponin I might be a useful biomarker for cardiac muscle involvement.

Heart transplantation may be the most effective intervention [30]. The only female patient who underwent heart transplantation was a 42-year-old woman with dilated cardiomyopathy 990 days after the LVAD operation [17]. For one year after the transplantation, she had no clinically significant deterioration. As cardiomyopathy can be fatal in both males and females, we should consider heart transplantation in Danon disease. However, due to the shortage of donor hearts in Japan, patients must wait three years until they can undergo heart transplantation. Therefore, an LVAD may help patients survive to transplantation.

In addition, we should consider investigating asymptomatic female relatives of male patients for cardiomyopathy and carefully follow-up such patients to detect initial symptoms of a potentially life-threatening condition. Interestingly, in the present survey, we identified six young female patients who received a diagnosis of Danon disease, the cardiac symptoms of which usually develop in adulthood in females. One patient had very early onset at the age of six years, with severe cardiac failure similar to that in males [22]. Globally, very few female patients with disease onset in childhood have been described previously [31,32,33]. However, these female patients had different phenotypes with respect to the severity of the cardiomyopathy. Concerning cardiomyopathy, Oldfors et al. proposed that an uneven distribution of the LAMP-2 protein might cause the dysfunction of cardiomyocytes [31]. In contrast, Maron et al. reported that many autophagic vacuoles were scattered in the cardiomyocytes [32]. Moreover, we previously reported that the number of muscle fibers with an accumulation of autophagic vacuoles increase in proportion to age [8]. On the basis of these findings, we suggest that the progression of clinical symptoms is related not only to primary deficiency of LAMP-2, but also to the accumulation of autophagic vacuoles increasing with age, and that the accumulation of autophagic vacuoles probably contributes to pathogenesis of the disease. Therefore, we hypothesize that the clinical presentations of Danon disease depend directly on not only LAMP-2 deficiency, but also on the accumulation of autophagic vacuoles.

Myopathy was usually mild and can be clinically silent; however, most male patients had elevated serum CK levels, myogenic changes on the EMG, or both. Symptomatic male patients showed proximal limb weakness, which was very slowly progressive or stable. In contrast, myopathy was observed in only a few female patients and was even milder.

Although mental retardation was observed in 46% of male patients and 9% of female patients, many patients had quite mild symptoms. Actually, some patients who were subject to a psychological examination revealed a mild learning disability and intellectual disability. In addition, their learning disturbance did not progress, and their condition remained stable [9]. The EEG showed mild abnormalities in only two male patients. There were no central nervous system (CNS) manifestations, and no patient showed structural brain abnormalities on magnetic resonance imaging or single photon emission computed tomography in the present survey [9]. A recent study suggested that LAMP-2 in the CNS might play a role in the degradation of intracytoplasmic molecules in lysosomes and protection from oxidative stress in the midbrain in LAMP-2-deficient mice [34]. However, the association between the CNS pathology and mental retardation remains unclear and unestablished. Therefore, we suggest that further neuropathologic analyses are needed to characterize the CNS involvement in Danon disease.

Retinopathy has been regarded as one of the complications of Danon disease [35]. Actually, in the present survey two patients (10%) had retinopathy. However, we should consider the possibility of an underdiagnosis of retinopathy due to very mild subjective symptoms. Moreover, retinopathy could potentially be used to identify asymptomatic patients.

Cerebral infarction is quite a rare complication of Danon disease. Previously four patients in whom cerebral infarction developed were reported [36]. Three of these patients had cardioembolic strokes caused by atrial fibrillation. The other patient showed a hemodynamic mechanism due to hypoperfusion. In the present survey, minor right cerebral infarction developed after the LVAD operation in one of the two female patients with cerebral infarction [17]. The other patient showed young-onset stroke due to diffuse narrowing of the cerebral arteries without atherosclerosis. Recently, based on the detailed analysis of this Japanese patient and LAMP-2-deficient mice, we found arterial medial hypertrophy with the phenotypic conversion of vascular smooth muscle cells, resulting from the age-dependent accumulation of cellular waste generated by aberrant autophagy [19]. Further studies including pathological evaluations should be performed to establish the pathomechanism of vasculopathy in cerebral infarction associated with Danon disease.

Muscle pathology was characterized by AVSF in the muscle fibers of male patients using light microscopy and electron microscopy. AVSF with acetylcholinesterase activity comprised the unique disease-specific autophagic vacuoles that delineate Danon disease from related myopathies [6]. LAMP-2 was completely absent in muscle from all male patients. In all male patients, acetylcholine and nonspecific esterase activity were associated with the vacuolar membranes.

In contrast, female patients showed normal staining of acetylcholinesterase and non-specific esterase and no AVSF in biopsy muscle specimens. On immunohistochemical and Western blot analyses of muscle specimens, two female patients showed a decreased, but not an absence of LAMP-2 expression [8,9]. Therefore, we attributed these results to a 50% reduction in LAMP-2, i.e., ‘LAMP-2 haploinsufficiency’, caused by a heterozygous null mutation in *LAMP-2* in female patients [5]. In fact, our findings suggest that a 50% reduction in LAMP-2 may prevent the development of myopathy, but not cardiomyopathy. Female patients show milder and later-onset symptoms than male patients [1,2]. As for the underlying reason, we suggest that LAMP-2 haploinsufficiency along with X-chromosome inactivation are one of the most likely important determinants of phenotype in female patients. However, Bottillo et al. reported that immunohistochemistry for LAMP2 revealed a mosaic pattern of distribution in the explanted heart muscles and suggested that this finding paralleled X chromosome inactivation within the cardiac muscles, but not skeletal muscles [37]. Further studies including more detailed pathological evaluations simultaneously in the cardiac and the skeletal muscles should be performed to establish the X chromosome inactivation in female patients with Danon disease.

In the sequence analysis of *LAMP-2*, only two families had the same mutation, while the other families had 17 different mutations, respectively. The international report on Danon disease also showed many kinds of mutations and a wide distribution of mutations ranging from exon 1 to exon 9, consistent with our study [30]. Interestingly, the mothers of the probands from 10 families had no cardiac involvement and no mutations in *LAMP-2*. Of the 10 families, we suggest that germline mosaicism was indicated in the two families in whom the probands’ siblings were also affected, as reported previously [10,16]. Therefore, we considered that the other eight (40%) of all 20 families had de novo mutations and that this finding was one of the genetic features of Danon disease. In addition, of 20 families, 16 probands were males, and four probands were females. All four female probands had de novo mutations. Therefore, we should consider the possibility that a female patient without a family history can be given a diagnosis of Danon disease.

On the other hand, human exon 9 of *LAMP-2* exists in two forms, exon 9a and 9b, which are alternatively spliced and produce two isoforms, LAMP-2a and LAMP-2b [1]. LAMP-2a is present ubiquitously, whereas LAMP-2b is expressed predominantly in heart and skeletal muscles. The mutation in Family 1 affects only the LAMP-2b isoform, whereas all other mutations affect both the LAMP-2a and 2b isoforms. The two affected males in Family 1 had hypertrophic cardiomyopathy and mild myopathy, but no mental retardation. In this family, one male patient who had mild cardiomyopathy, but reported no cardiac symptoms is alive at the age of 50 years, although no male patient survived beyond the age of 26 years except for in this family. In addition, the proband in Family 1 revealed a small amount of LAMP-2 protein on immunoreactivity studies, as reported previously [1]. Therefore, we suggest that the isolated deficiency of the LAMP-2b isoform may be associated with a milder phenotype, although we could not find a genotype–phenotype correlation, except for exon 9. Moreover, we suspect that genetic factors including genotype–phenotype correlation might be one of the most significant determinants of the unusual presentation of clinical features. The establishment of the exact mechanisms leading to the different phenotypes in Danon disease requires further investigation.

Danon disease is primarily caused by loss-of-function mutations in the *LAMP-2* gene. However, the exact pathomechanism of Danon disease is still not completely understood. LAMP-2 is a single transmembrane protein in the limiting membranes of lysosomes. Most mutations of the gene lead to protein truncation or splicing defects, resulting in a loss of transmembrane and/or cytoplasmic domain, leading to LAMP-2 protein deficiency. Although the precise functional role of LAMP-2 remains controversial, LAMP-2 protein is a key molecule in the final process of autophagy that mediates the fusion between autophagosomes and lysosomes. Therefore, we suggest that LAMP-2 deficiency leads to a failure in macroautophagy and may be related to the development of autophagic vacuoles, especially the AVSF.

In conclusion, Danon disease may be caused by primary lysosomal dysfunctions and is an extremely rare muscular disorder in Japan. Cardiomyopathy is the most significant prognostic factor and the main cause of death. However, Danon disease may be overlooked in patients who have hypertrophic cardiomyopathy, since other clinical features including myopathy can be mild, particularly in females. Our findings suggest that the present survey can extend our understanding of the clinical features of patients, especially female patients, with this rare disease.

## 4. Materials and Methods

### 4.1. Nationwide, Questionnaire-Based Survey

A nationwide survey on Danon disease was conducted using two-step questionnaires to elucidate its prevalence, clinical features, muscle pathological findings, and mutation status in Japan. The first step of questionnaires focused on the experience of the previous and current patients with definite Danon disease and treated patients suspected to have Danon disease. The first set of questionnaires was sent to a total of 2617 clinical departments with board-certified specialists in neurology, cardiology, and pediatrics in Japan. The second step of questionnaires, which focused on the clinical information of patients with definite and suspected Danon disease, was sent to the departments that their attending physicians belonged to. When a patient was suspected of having Danon disease, genetic analyses of the LAMP-2 gene were performed. In addition, we carefully reviewed the medical records and clinical histories. Statistical values are expressed as mean ± standard deviation. All clinical materials used in this study were obtained for diagnostic purposes with written informed consent. All surveys and experiments performed in this study were approved by the Ethical Committee of Nara Medical University (approval nos. 926 (20 October 2014)).

### 4.2. Histochemistry and Immunohistochemistry

All biopsy specimens were taken from the patients’ muscles. These specimens were frozen in liquid-nitrogen-cooled isopentane for histochemical and immunohistochemical assessment. Transverse frozen sections of 8-μm-thickness were stained with hematoxylin and eosin and a battery of histochemical methods. In addition, serial cryosections of 6-μm-thickness were stained with mouse monoclonal antibodies, as described previously [6]. These sections were incubated at 37 °C for 2 h with primary mouse monoclonal IgG antibodies against LAMP-2 (H4B4, Developmental Studies Hybridoma Bank (DSHB), Iowa, IA, USA), lysosomal integral membrane protein-1 (LIMP-1) (H5C6, DSHB), the N-terminus of dystrophin (NCL-DYS3, Novocastra, Newcastle Upon Tyne, UK), α-sarcoglycan (NCL-L-a-SARC, Novocastra), and laminin α2 (MAB1922, Chemicon, Temecula, CA, USA). Subsequently, these sections were incubated at room temperature for 1 h with a secondary antibody, goat anti-mouse IgG (M111, Leinco, St. Louis, MO, USA). Control specimens were taken from six patients with morphologically normal muscle.

### 4.3. Electron Microscopy

For the electron microscopy, one portion of the biopsied muscle specimens was fixed in buffered 2% isotonic glutaraldehyde at pH 7.4 and then post-fixed in osmium tetroxide. Subsequently, one fragment of the specimens was embedded in epoxy resin. Ultrathin sections were stained with uranyl acetate and lead citrate, as described previously [6]. We examined these sections with an H-7000 electron microscope (Hitachi, Tokyo, Japan).

### 4.4. Western Blot Analysis of Muscle Specimens

Protein lysates for Western blot analysis were prepared from muscle specimens from some male and two female patients with Danon disease, as well as from male and female controls, as described previously [8,9]. We briefly washed the frozen tissue specimens with cold phosphate-buffered saline, homogenized in triple-detergent lysis buffer, and spun them down. After the supernatant was collected, we separated 5-μl samples by electrophoresis and subsequently electro-transferred the proteins onto nitrocellulose membranes. The membranes were incubated with a primary mouse monoclonal IgG antibody against LAMP-2 (DSHB). Quantitative measurements of the resulting images were analyzed using densitometry. In the summarized results, we used the ratio of the expression values of LAMP-2 to β-actin. Relative protein expression levels in the male controls were expressed as 100%.

### 4.5. Sequence Analysis of LAMP-2

For the sequence analysis, genomic DNA was extracted from either frozen muscle or peripheral blood lymphocytes with phenol/chloroform. The open reading frame of *LAMP-2* consists of nine exons. Human exon 9 exists in two forms, exon 9a and 9b, which are alternatively spliced and processed into two isoforms, LAMP-2a and LAMP-2b. We sequenced the entire coding region, including both exons 9a and 9b, and the exon/intron junctions of *LAMP-2*, as described previously [1,2].

## Figures and Tables

**Table 1:** Summary of patients with Danon disease in Japan.

Family	Site of *LAMP-2* Mutation	*LAMP-2* Mutation	Effect on mRNA	Affected Males	Affected Females	References
1	E9b	c.1097_1098 delAA	Frame shift	proband, 1 cousin	mother, 1 sister	[2]
2	E4	c.440 T > A	Stop codon	proband	-	[2]
3	I6	c.865-1 G > C	Splicing	proband	mother	[2]
4	I5	c.741+1G>A	Stop codon	proband	-	[2]
5	I5	c.742-4_742+6 delGAAGGTTGCT	Splicing	proband	mother	[2,8]
6	E7	c.883_884 insT	Frame shift	proband	1 sister	[2,10]
7	E3	c.288_289 delAG	Stop codon	proband	mother, 1 aunt	[11]
8	E7	c.928 G>A	Splicing	proband	-	[12]
9	E5	c.573 delA	Frame shift	proband	mother, 2 sisters	[13]
10	E7	c.877 C>T	Stop codon	proband	mother, 1 sister	[14]
11	E3	c.371_372 delCA	Stop codon	proband	-	[15]
12	I6	c.864+1_864+4 delGTGA	Splicing	proband	2 sisters	[16]
13	E6	c.749C>A	Stop codon	-	proband	[9]
14	E7	c.877C>T	Stop codon	proband	mother	[17]
15	E1	c.56T>C	Frame shift	proband	mother	[18]
16	E8	c.1009_1010delGT	Frame shift	proband	mother	[19]
17	NA	NA	NA	proband	mother	[20]
18	E8	c.973_974insC	Frame shift	-	proband	[21]
19	E4	c.422T>A	Stop codon	-	proband	[22]
20	E6	c.839delA	Stop codon	-	proband	[17]

E, exon; I, intron; NA, not available.

**Table 2:** Clinical characteristics of patients with Danon disease in Japan.

Characteristics	Male	Female
Subjects, n	17	22
Probands, n	16	4
Manifesting mothers of the probands, n (%)	10/16 (63%)	0/4 (0%)
Age at onset, n		
Infantile	4	0
Childhood	9	1
Second decade	4	5
Adult	0	1
Unknown	0	15
Alive at the time of the survey, n	7	11
Dead at the time of the survey, n	10	11
Age at death, year-old, mean ± SD	19 ± 5 (n = 7)	37 ± 11 (n = 7)
Cause of death, n (%)		
Cardiac failure	9/10 (90%)	11/11 (100%)
Cancer	1/10 (10%)	0/0 (0%)
Myopathy, n (%)	17/17 (100%)	2/22 (9%)
Muscle weakness	13/17 (76%)	2/22 (9%)
No weakness with myogenic change on EMG	4/17 (24%)	0/22 (0%)
Cardiomyopathy, n (%)	17/17 (100%)	21/22 (95%)
Hypertrophic	15/17 (88%)	11/22 (50%)
Dilated	2/17 (12%)	6/22 (27%)
Unknown	0/17 (0%)	4/22 (18%)
Mental retardation, n (%)	8/17 (46%)	2/22 (9%)
Retinopathy, n (%)	1/17 (6%)	1/22 (5%)
Pes cavus, n (%)	1/17 (6%)	1/22 (5%)
Cerebral infarction, n (%)	0/17 (0%)	2/22 (9%)
Heart transplantation, n (%)	0/17 (0%)	1/22 (5%)
Awaiting heart transplantation, n (%)	2/17 (12%)	3/22 (14%)
Left ventricular assist device, n (%)	1/17 (6%)	4/22 (18%)
Treatment of blocker, n (%)	6/17 (35%)	11/22 (50%)
Elevated CK, n (%)	16/17 (94%)	3/12 (25%)
Serum CK (IU/L), mean ± SD	1086 ± 715	148 ± 164
Abnormal ECG, n (%)	17/17 (100%)	18/18 (100%)
WPW syndrome, n (%)	9/17 (54%)	4/18 (22%)
Abnormal echocardiogram, n (%)	17/17 (100%)	17/18 (94%)
Myogenic EMG, n (%)	11/11 (100%)	0/6 (0%)
Abnormal nerve conduction study, n (%)	1/12 (8%)	0/1 (0%)

SD, standard deviation; EMG, electromyography; CK, creative kinase; ECG, electrocardiogram; WPW, Wolff–Parkinson–White.

## Abbreviations

AVSF	autophagic vacuoles with sarcolemmal features
CK	creative kinase
CNS	central nervous system
EEG	electroencephalography
ECG	electrocardiogram
LAMP-2	lysosome-associated membrane protein-2
LIMP-1	lysosomal integral membrane protein-1
LVAD	left ventricular assist devices
WPW	Wolff–Parkinson–White

## References

[1] Nishino I., Fu J., Tanji K., Yamada T., Shimojo S., Koori T., Mora M., Riggs J.E., Oh S.J., Koga Y. (2000). Primary LAMP-2 deficiency causes X-linked vacuolar cardiomyopathy and myopathy (Danon disease). Nature.

[2] Sugie K., Yamamoto A., Murayama K., Oh S.J., Takahashi M., Mora M., Riggs J.E., Colomer J., Iturriaga C., Meloni A. (2002). Clinicopathological features of genetically confirmed Danon disease. Neurology.

[3] Danon M.J., Oh S.J., DiMauro S., Manaligod J.R., Eastwood A., Naidu S., Schliselfeld L.H. (1981). Lysosomal glycogen storage disease with normal acid maltase. Neurology.

[4] Sugie K., Nishino I., Rosenberg R.N., Pascual J.M. (2014). Lysosomal Membrane Disorders: LAMP-2 Deficiency. Rosenberg’s Molecular and Genetic Basis of Neurological and Psychiatric Disease.

[5] Nishino I., Yamamoto A., Sugie K., Hirano M., Nonaka I. (2001). Danon disease and related disorders. Acta Myologica.

[6] Sugie K., Noguchi S., Kozuka Y., Arikawa-Hirasawa E., Tanaka M., Yan C., Saftig P., von Figura K., Hirano M., Ueno S. (2005). Autophagic vacuoles with sarcolemmal features delineate Danon disease and related myopathies. J. Neuropathol. Exp. Neurol..

[7] Endo Y., Furuta A., Nishino I. (2015). Danon disease: A phenotypic expression of LAMP-2 deficiency. Acta Neuropathol..

[8] Sugie K., Koori T., Yamamoto A., Ogawa M., Hirano M., Inoue K., Nonaka I., Nishino I. (2003). Characterization of Danon disease in a male patient and his affected mother. Neuromuscul. Disord..

[9] Sugie K., Yoshizawa H., Onoue K., Nakanishi Y., Eura N., Ogawa M., Nakano T., Sakaguchi Y., Hayashi Y.K., Kishimoto T. (2016). Early onset of cardiomyopathy and intellectual disability in a girl with Danon disease associated with a de novo novel mutation of the LAMP-2 gene. Neuropathology.

[10] Takahashi M., Yamamoto A., Takano K., Sudo A., Wada T., Goto Y., Nishino I., Saitoh S. (2002). Germline mosaicism of a novel mutation in lysosome-associated membrane protein-2 deficiency (Danon disease). Ann. Neurol..

[11] Tada H., Harimura Y., Yamasaki H., Sekiguchi Y., Ishizu T., Seo Y., Kawano S., Aonuma K. (2010). Utility of real-time 3-dimensional echocardiography and magnetic resonance imaging for evaluation of Danon disease. Circulation.

[12] Fukushima H., Yamagishi T., Dobashi T., Nakazawa M., Hayashi T., Furumichi K. (2004). A case of Danon disease with paroxysmal supraventricular tachycardia (translation). Pediatr. Cardiol. Card. Surg..

[13] Dougu N., Joho S., Shan L., Shida T., Matsuki A., Uese K., Hirono K., Ichida F., Tanaka K., Nishino I. (2009). Novel LAMP-2 mutation in a family with Danon disease presenting with hypertrophic cardiomyopathy. Circ. J..

[14] Awaya T., Yoshida T., Shibata M., Kato T. (2012). Flowcytometric diagnosis of Danon disease (LAMP-2 Deficiency) (translation). No to Hattatsu.

[15] Tamura T., Nishida K. (2010). A case of Danon disease diagnosed by hypertrophic cardiomyopathy complicated with eye lesions (translation). Pediatr. Cardiol. Card. Surg..

[16] Hashida Y., Wada T., Saito T., Ohta K., Kasahara Y., Yachie A. (2015). Early diagnosis of Danon disease: Flow cytometric detection of lysosome-associated membrane protein-2-negative leukocytes. J. Cardiol..

[17] Kitahara H., Nawata K., Kinoshita O., Itoda Y., Shintani Y., Fukayama M., Ono M. (2017). Implantation of a Left Ventricular Assist Device for Danon Cardiomyopathy. Ann. Thorac. Surg..

[18] Namatame S., Segawa M., Matsuda N., Hoshi A., Ugawa Y. (2015). A case of Danon disease (translation). Rinsho Shinkeigaku.

[19] Nguyen H.T., Noguchi S., Sugie K., Matsuo Y., Nguyen C.T.H., Koito H., Shiojima I., Nishino I., Tsukaguchi H. (2018). Small-Vessel Vasculopathy Due to Aberrant Autophagy in LAMP-2 Deficiency. Sci. Rep..

[20] Takumi Y., Akagi T., Hiratsuka T., Shibata T., Ueda T., Tojigamori M., Shiroshita H., Etoh T., Inomata M., Noguchi T. (2015). Laparoscopic transverse colon resection in a patient of juvenile transverse colon cancer with hypertrophic cardiomyopathy due to Danon disease (translation). J. Jpn. Surg. Assoc..

[21] Nagatomo Y., Kuraoka A., Muneuchi J., Terashi E., Sugitani Y., Takenaka S., Watanabe M., Shiroo K. (2014). A girl patient of Danon disease diagnosed because of school cardiac examination (translation). J. Jpn. Pediatr. Soc..

[22] Yamamoto K., Ootsuka Y., Miyake K., Takahashi K., Nakayashiro M., Takakuwa H., Fukushima N., Ichida F., Nishino I. (2013). A girl patient of Danon disease with a novel LAMP-2 mutation diagnosed by severe heart failure (translation). J. Jpn. Pediatr. Soc..

[23] Maron B.J., Roberts W.C., Arad M., Haas T.S., Spirito P., Wright G.B., Almquist A.K., Baffa J.M., Saul J.P., Ho C.Y. (2009). Clinical outcome and phenotypic expression in LAMP-2 cardiomyopathy. JAMA.

[24] Yang Z., McMahon C.J., Smith L.R., Bersola J., Adesina A.M., Breinholt J.P., Kearney D.L., Dreyer W.J., Denfield S.W., Price J.F. (2005). Danon disease as an underrecognized cause of hypertrophic cardiomyopathy in children. Circulation.

[25] Arad M., Maron B.J., Gorham J.M., Johnson W.H., Saul J.P., Perez-Atayde A.R., Spirito P., Wright G.B., Kanter R.J., Seidman C.E. (2005). Glycogen storage diseases presenting as hypertrophic cardiomyopathy. N. Engl. J. Med..

[26] Riggs J.E., Schochet S.S., Gutmann L., Shanske S., Neal W.A., DiMauro S. (1983). Lysosomal glycogen storage disease without acid maltase deficiency. Neurology.

[27] Mahdhaoui A., Bouraoui H., Tabarki B., Majdoub M., Trimeche B., Mahdhaoui N., Chabrak S., Ernez-Hajri S., Jeridi G., Ammar H. (2003). Familial hypertrophic cardiomyopathy associated with Wolff-Parkinson-White syndrome. Acta. Clin. Belg..

[28] Marriott H.T.L. (1964). Electrocardiographic abnormalities, conduction disorders and arrhythmias in primary myocardial disease. Prog. Cardiovasc. Dis..

[29] Roos J.C.P., Daniels M.J., Morris E., Hyry H.I., Cox T.M. (2018). Heterogeneity in a large pedigree with Danon disease: Implications for pathogenesis and management. Mol. Genet. Metab..

[30] Boucek D., Jirikowic J., Taylor M. (2011). Natural history of Danon disease. Genet. Med..

[31] Hedberg Oldfors C., Máthé G., Thomson K., Tulinius M., Karason K., Östman-Smith I., Oldfors A. (2015). Early onset cardiomyopathy in females with Danon disease. Neuromuscul. Disord..

[32] Kim H., Cho A., Lim B.C., Kim M.J., Kim K.J., Nishino I., Hwang Y.S., Chae J.H. (2010). A 13-year-old girl with proximal weakness and hypertrophic cardiomyopathy with Danon disease. Muscle Nerve.

[33] Dworzak F., Casazza F., Mora M., De Maria R., Gronda E., Baroldi G., Rimoldi M., Morandi L., Cornelio F. (1994). Lysosomal glycogen storage with normal acid maltase: A familial study with successful heart transplant. Neuromuscul. Disord..

[34] Furuta A., Kikuchi H., Fujita H., Yamada D., Fujiwara Y., Kabuta T., Nishino I., Wada K., Uchiyama Y. (2015). Property of lysosomal storage disease associated with midbrain pathology in the central nervous system of Lamp-2-deficient mice. Am. J. Pathol..

[35] Prall F.R., Drack A., Taylor M., Ku L., Olson J.L., Gregory D., Mestroni L., Mandava N. (2006). Ophthalmic manifestations of Danon disease. Ophthalmology.

[36] Marino M., Musumeci O., Paleologo G., Cucinotta M., Migliorato A., Rodolico C., Toscano A. (2016). Ischemic stroke due to hypoperfusion in a patient with a previously unrecognized Danon disease. Neuromuscul. Disord..

[37] Bottillo I., Giordano C., Cerbelli B., D’Angelantonio D., Lipari M., Polidori T., Majore S., Bertini E., D’Amico A., Giannarelli D. (2016). A novel LAMP2 mutation associated with severe cardiac hypertrophy and microvascular remodeling in a female with Danon disease: A case report and literature review. Cardiovasc. Pathol..

